# Medical Reform in Spain

**Published:** 1836-04

**Authors:** 


### MEDICAL REFORM IN SPAIN.

In the *Boletin de Medicina, Cirujia y Farmacia*, No. 75, tom. ii. 1835, Jueves, 5 de Noviembre, is a royal decree, dated Nov. 1, 1835, constituting a commission of five persons and a secretary, (among whom we observe the name of our friend and correspondent, Dr. Mateo Seoane, "vocal de la junta suprema de Sanidad,") for the purpose of "examining and proposing such modifications as the existing laws are susceptible of." The following is the preamble of the decree: "Animated by pure zeal for the preservation of the public health; convinced of the necessity of rendering uniform the studies which are the basis of the healing art, in all the universities and colleges which now exist or may hereafter exist; and persuaded of the advantage of duly honouring those who dedicate themselves to a profession as noble as necessary; I have thought well to institute a commission which may examine and propose to me such modifications as the laws now in force may be susceptible of, and whatever else may contribute to its lustre and welfare."

